# Seasonality Patterns of Internet Searches on Mental Health: Exploratory Infodemiology Study

**DOI:** 10.2196/12974

**Published:** 2019-04-24

**Authors:** Noam Soreni, Duncan H Cameron, David L Streiner, Karen Rowa, Randi E McCabe

**Affiliations:** 1 Anxiety Treatment and Research Clinic St. Joseph's Healthcare Hamilton Hamilton, ON Canada; 2 Department of Psychiatry and Behavioural Neurosciences McMaster University Hamilton, ON Canada

**Keywords:** anxiety, depression, OCD, schizophrenia, autism, suicide, seasonality, Google, internet, infodemiology, infoveillance, mental health

## Abstract

**Background:**

The study of seasonal patterns of public interest in psychiatric disorders has important theoretical and practical implications for service planning and delivery. The recent explosion of internet searches suggests that mining search databases yields unique information on public interest in mental health disorders, which is a significantly more affordable approach than population health studies.

**Objective:**

This study aimed to investigate seasonal patterns of internet mental health queries in Ontario, Canada.

**Methods:**

Weekly data on health queries in Ontario from Google Trends were downloaded for a 5-year period (2012-2017) for the terms “schizophrenia,” “autism,” “bipolar,” “depression,” “anxiety,” “OCD” (obsessive-compulsive disorder), and “suicide.” Control terms were overall search results for the terms “health” and “how.” Time-series analyses using a continuous wavelet transform were performed to isolate seasonal components in the search volume for each term.

**Results:**

All mental health queries showed significant seasonal patterns with peak periodicity occurring over the winter months and troughs occurring during summer, except for “suicide.” The comparison term “health” also exhibited seasonal periodicity, while the term “how” did not, indicating that general information seeking may not follow a seasonal trend in the way that mental health information seeking does.

**Conclusions:**

Seasonal patterns of internet search volume in a wide range of mental health terms were observed, with the exception of “suicide.” Our study demonstrates that monitoring internet search trends is an affordable, instantaneous, and naturalistic method to sample public interest in large populations and inform health policy planners.

## Introduction

There is emerging evidence on the existence of seasonal patterns of public interest in psychiatric disorders and conditions [1]. The increasing number of studies on the topic is associated with several factors such as the identification of seasonal patterns of population behavior in humans [2,3] and animals [4] and the realization that understanding temporal variation of public interest in health-related issues has important theoretical as well as practical implications on service planning and delivery [5-7]. The terms "infodemiology" or "infoveillance" were coined to describe novel methods to analyze search and publication behavior (for example, tweets) on the Internet to study trends and human behaviour to inform public health practice and policy [6]. For example, the availability of up to date information on internet searches may lead to radical rethinking of the need to reduce the optimal interval between service planning and delivery [6] and affect the frequency by which public health policies are updated. There is ample evidence that psychiatric disorders and symptoms often have seasonal patterns [8]: Seasonal affective disorder [9], in which depressive symptoms worsen during a typical time each year, is perhaps the best example of seasonal changes in psychiatric symptoms. In addition, studies have reported seasonality patterns for anxiety disorders [10], obsessive-compulsive disorder (OCD) [10], and psychotic disorders [11]. Most recently, evidence showed that seasonality patterns are similar across different climate zones [1].

Until recently, the sampling of populations for information on mental health was mostly conducted using epidemiological surveys, which have been widely used to study evidence on the level of disorders in the general population [12,13]. However, the majority of epidemiological surveys involve a compromise between the need to acquire frequent measurements and lengthy follow-up periods, whereas the study of seasonality effects requires high-resolution and accurate time-series measurements. Furthermore, population surveys are costly and limited by respondents’ reticence associated with privacy and stigma. For example, respondents are reluctant to report data that are associated with social stigma [14] or may expose their income [15]. Finally, there is evidence of progressive, constant reduction in response rates to epidemiologic public health studies [16].

Limitations of existing epidemiological surveys are associated with a dearth of knowledge surrounding the possible existence of seasonal changes in public interest of psychiatric disorders and conditions. This knowledge gap is of importance, given the growing recognition of public interest in mental health and psychiatric issues [17], the need for effective population informatics, and limited temporal resolution of existing clinical databases or traditional epidemiological surveys [18].

Over the last two decades, progress has been made in our ability to assess public interest in mental health issues by tracking internet searches. The internet became the most relied upon health search resource as early as 2006 [19], and there is evidence that internet search volumes are associated with real-time markers of illness [6,20]. Internet searches for health information appear to be more common in individuals who experience difficulty accessing health care services [21]. There is evidence that individuals with a history of mental health issues are more likely to use the internet for mental health searches than those without any mental health issues [22]. Thus, tracking internet search queries may represent an advance in our ability to monitor continuous population trends related to mental health issues [1]. However, although recent studies recognize the potential of internet search studies to address existing gaps in the epidemiological literature [23,24], the field is still in its early stages.

Internet searches are most frequently performed using the Google search engine. In 2017, Google search accounted for 67.5% of overall searches in Canada, over three times the number of Yahoo searches (21.5%) [25]. A recent study of Google mental health queries in the United States and Australia suggested the presence of seasonal patterns of mental health searches [1]. Specifically, the authors reported that winter peaks and summer troughs accounted for 14% and 11% of the differences in search volumes for the United States and Australia, respectively. The authors reported that seasonal patterns were evident, with some variation, for specific mental health categories (ie, anxiety, attention-deficit-hyperactivity disorder, anxiety, bipolar disorder, depression, anorexia or bulimia, OCD, schizophrenia, and suicide). However, the study targeted two large, geographically and demographically diverse countries that cover opposite climate zones. There is at least preliminary evidence [26] that factors associated with climate (eg, temperature and evaporation) may be associated with seasonal variations in mental health problems, and there is a need to study the seasonality of mental health symptoms in smaller areas in a more homogenous climate.

To date, however, no studies of internet queries have examined aspects of seasonality of interest in psychiatric conditions in smaller geographical areas that are relatively homogenous with regard to climate. This exploratory study investigated seasonal patterns of Google queries on mental health diagnoses and symptoms in Ontario, Canada’s most populous province and home to over 13 million individuals. Given the lack of existing research in this field and the inability to record specific population demographic information, the sample was limited to Ontario in order to reduce the possible influence of different climate zones.

## Methods

### Data Collection

Data were downloaded from Google Trends [27], the public database of Google queries. Google Trends presents data as a relative search volume in a normalized format. The period with the highest proportion of searches related to the key term within a category receives a value of 100, and a value of 50 is 50% of that maximum proportion, that is, the point over the course of the selected period (5 years in this study) at which the search volume is greatest for a search term is provided a value of 100, and the remaining values are assigned as a proportion of the points of the maximum search volume. Google trends allows extraction of data for only a single search term at a time and does not provide absolute values of search data.

English search terms were captured from Google Trends data in Ontario for the 5-year period from August 2012 to August 2017. For the purpose of this study, primary search terms were similar to those chosen by Ayers and colleagues [1]—“anxiety,” “autism,” “bipolar,” “depression,” “OCD,” “schizophrenia,” and “suicide”—these terms were then run within and downloaded from Google Trends’ mental health category.

We measured seasonality of search interest in general aspects of health by downloading search results for the term “health” in the general health category. To measure the seasonality of even broader search interests that extend well beyond health-related issues, we downloaded search results for the content-agnostic term “how” that can be used as an adverb, a conjunction, or a noun. Due to the paucity of research in this area, these search terms were not based on previous study but were rather decided upon based on consensus opinion between the authors.

Using Google Trends’ related-term option, we extracted the top 10 related searches for each of our items in their respective categories. We then excluded searches that were ostensibly unrelated to the question of this study (eg, the term “Suicide Squad” denoting a blockbuster movie rather than a mental health query). Terms that included overlap between search terms (eg, “anxiety and depression”) were excluded from both search lists. Thereafter, the original search term (eg, “bipolar”) and the remaining related searches were used to calculate the mean value for the weekly data point of the 5-year time series.

### Data Analysis

#### Seasonal Components

Our primary aim was to assess whether a significant seasonal signature was detected for each of the search terms across a 5-year period. Using the R package WaveletComp [28], separate continuous wavelet transform analyses were performed to explore periodicity in the 5-year time series for each search term. A wavelet transform is a function which, in this study, was used to divide a continuous time series signal into smaller components (wavelets) and then examine the intensity and timing of the seasonal periodicity of the original time series. The process is similar to a Fourier transform but can effectively increase the signal-to-noise ratio, providing a more accurate representation of the trend underlying the time series [29]. In this study, the wavelet transform was used for smooth high-resolution time series data (ie, weekly data points over a 5-year period) to reduce noise, allowing for more clear observation of trends in the data over time. This method can be applied to any high-resolution data over time to decompress the signal (or data) in order to observe trends over time. Some other applications of the wavelet transform include image processing (eg, neuroimaging), pattern recognition, or noise reduction of any waveform data (eg, electroencephalogram signal).

Each series was decomposed in the time-frequency domain using a continuous wavelet transform. The resulting wavelet power spectrum was used to identify whether a significant 52-week periodic component was detected (*P*<.05). If no significant seasonal pattern was detected for a particular search term, no further analysis was performed for that term. The coefficients for each wave function were then extracted and plotted.

#### Seasonal Pattern Differences

Phase angle differences were calculated to assess timing differences between the wave functions of our search terms. Phase angle (measured from peak to peak) is the angular position along a sinusoidal function from –180º to +180º, where these extreme values represent two waves that are completely out of phase, while 0º would represent waves that are completely in phase. In the case of this study, with a 52-week periodicity, a phase shift of ±180º would indicate that the peaks of two waves being compared (ie, peak search volume for two search terms) are occurring exactly 6 months apart, with a positive angle indicating that the second wave shifted later in the year and a negative value indicating that the second wave shifted earlier in the year, relative to the first wave. Phase angle difference was also calculated within each search term, across each of the 5 years in order to assess a shift in the peak search volume from year to year. In order to translate the phase angle difference value into approximate weeks of the year, the value in degrees was divided by 360º and multiplied by 52.

Finally, we measured differences in the magnitude of seasonal changes between our search terms. First, percent change in search volume from summer to winter was calculated for each of the 5 years of the study. Means and SD of percent change from summer to winter in search volume were calculated for each term. We then performed a one-way analysis of variance, followed by posthoc tests, to assess differences between search terms in their percent change in search volume between August 2012 and August 2017. A one-way analysis of variance was chosen over multiple *t* tests to reduce the risk of type I error.

## Results

### Seasonal Components

**Figure 1:**
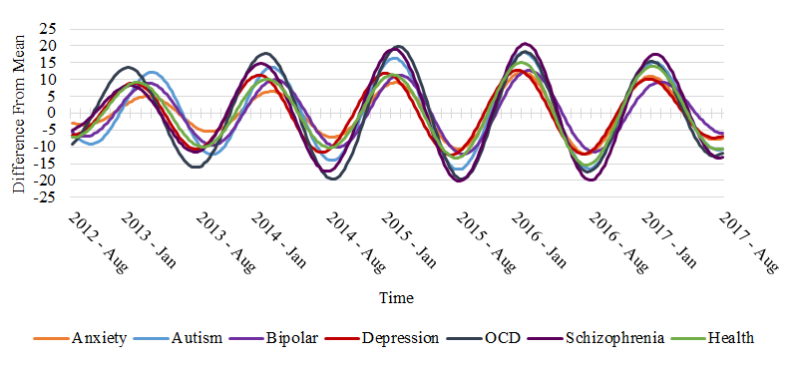
Change in relative search volume over time between search terms from August 2012 to August 2017. OCD: obsessive-compulsive disorder.

**Figure 2:**
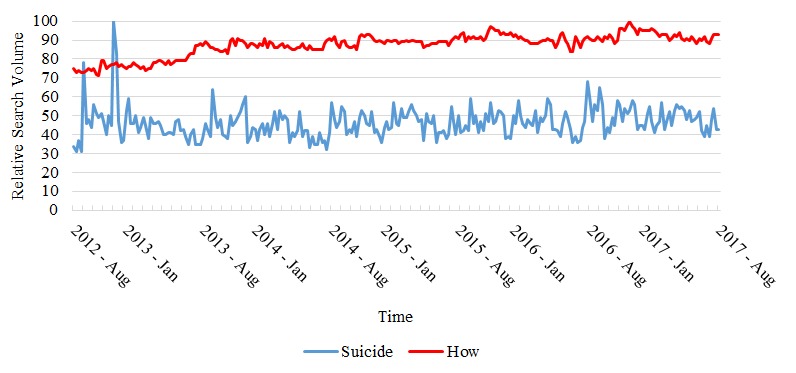
Nonseasonal search terms (raw data).

A significant 52-week seasonal component was found for all search terms, with the exception of “suicide” and our general control search term “how.” The waves for the terms showing a significant seasonal component are shown in Figure 1, and the raw data for search terms that showed no significant seasonality patters are featured in Figure 2. Significant seasonal components for all items had peak search volumes over the winter months and troughs during the summer months. Amplitude changes of search volume, as represented in the difference from the mean search volume for each year, showed an upward trend across the 5-year period for each of the searches. In other words, the seasonal variability in interest observed for each term tended to increase from year to year (with the exception of a slight decrease in 2017), and higher peaks were seen in winters and lower troughs were seen in summers over time.

### Magnitude of Seasonal Difference

Investigation of individual disorders showed that mean percent difference from winter to summer months was greatest for OCD (45.2%, SD 4.9%; 95% CI 40.8%-49.6%) and schizophrenia (43.3%, SD 10.5%; 95% CI 34.1%-52.5%). This indicates that the average 5-year peak search volumes for these terms were 45% and 43% higher in the winter than the lowest point in the summer, respectively. Autism also showed a similarly marked change in search volume, with a 37.5% difference from winter to summer (SD 6.6%; 95% CI 31.6%-43.4%). The differences for the remaining search terms were as follows: 21.2% for anxiety (SD 7.6%; 95% CI 14.6%-27.8%), 26.6% for bipolar disorder (SD 3.9%; 95% CI 23.5%-29.7%), and 28.7% for depression (SD 2.5%; 95% CI 26.5%-30.9%). Our term for searches related to “health,” in general, showed a mean seasonal change of 31.6% (SD 6.4%; 95% CI 26.0%-37.2%).

The one-way analysis of variance comparing the mean percent change in search volume between all search terms was significant (*F*_6,28_=9.04; *P*<.001; η^2^_p_=.693), indicating that the change in mean search volume from August 2012 to August 2017 was different between search terms. Results of the Tukey Honest Significant Difference posthoc comparisons are presented in Table 1. The greatest differences observed were for anxiety-schizophrenia (*P*<.001) and anxiety-OCD (*P*<.001), while the smallest differences were for bipolar-depression (*P*>.99) and OCD-schizophrenia (*P*>.99).

**Table 1:** Comparison of percent change in peak search volume and mean phase angle difference between terms across 5 years (August 2012 to August 2017). Phase angle values represent the difference in the timing of peak search volume between search terms across the 5-year search period. A positive phase angle difference represents a comparison in which the peak search volume for the second term is occurring later in the year relative to the first term, while a negative value indicates that peak volume for the second term occurs earlier in the year relative to the first. The value in parentheses is the value of the phase angle difference represented in weeks of the year.

Comparison	Mean difference (%)^a^	*P* value	Phase angle (degrees), weeks
Anxiety-autism	–16.2	.009	10.1 (1.5)
Anxiety-bipolar	–5.4	.85	14.6 (2.1)
Anxiety-depression	–7.5	.56	–22.3 (–3.2)
Anxiety-OCD^b^	–22.1	<.001	–7.5 (–1.1)
Anxiety-schizophrenia	–23.9	<.001	–10.8 (–1.6)
Anxiety-health	–10.4	.21	–21.0 (–3.0)
Autism-bipolar	10.8	.16	–4.5 (–0.7)
Autism-depression	8.7	.39	–36.9 (–5.3)
Autism-OCD	–5.9	.80	–22.1 (–3.2)
Autism-schizophrenia	–7.7	.53	–25.4 (–3.7)
Autism-health	5.8	.79	–16.7 (–2.4)
Bipolar-depression	–2.1	>.99	–32.4 (–4.7)
Bipolar-OCD	–16.7	.007	–17.6 (–2.5)
Bipolar-schizophrenia	–18.6	.002	–20.9 (–3.0)
Bipolar-health	–4.9	.89	–12.2 (–1.8)
Depression-OCD	–14.6	.02	14.8 (2.1)
Depression-schizophrenia	–16.4	.008	11.5 (1.7)
Depression-health	–2.8	.99	20.2 (2.9)
OCD-schizophrenia	–1.8	>.99	–3.3 (–0.5)
OCD-health	11.7	.11	11.5 (1.7)
Schizophrenia-health	13.6	.04	–5.4 (–0.8)

^a^Mean percent change in search volume between each pair of search terms are the results of the Tukey Honestly Significant Difference posthoc comparisons following one-way analysis of variance.

^b^OCD: obsessive-compulsive disorder.

### Timing Differences Between Search Terms

To assess differences in the timing for peak search volume between search terms across the 5-year period, the mean phase angle difference was estimated between all pairs of search terms (Table 1). Analysis of phase angle difference showed that the greatest difference in timing between any two search terms was between autism and depression, with a mean phase angle difference of –36.9º, corresponding to approximately 5.3 weeks, indicating that peak search volume occurred, on an average, 5.3 weeks earlier in the year for depression than for autism. The minimum phase angle difference was observed between “OCD” and “schizophrenia,” with a mean difference of –3.3º (or 0.48 weeks), indicating that these two searches were almost completely in phase, that is, peak search volume for these two terms occurred at almost exactly the same time for these two terms.

### Five-Year Shift in Peak Search Volume

**Figure 3:**
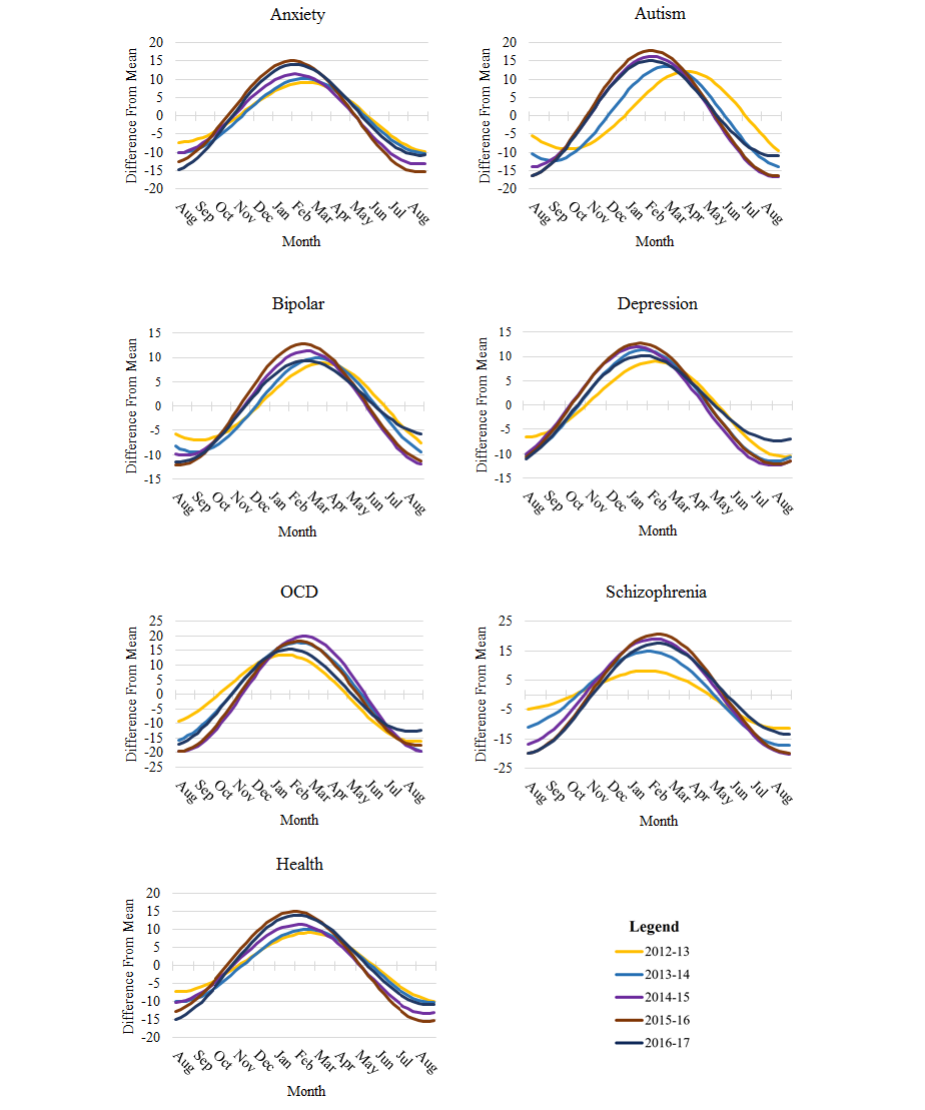
Peak search volume of search terms in different years. OCD: obsessive-compulsive disorder.

Phase angle difference was also calculated within each search term between consecutive years. As seen in Figure 3, there is a general negative shift in the search volume over the course of the 5-year period, indicating that peak search volume occurred slightly earlier in the winter months each year. The greatest year 1 to year 5 phase-angle shift was observed for autism, with a peak search volume occurring 7.5 weeks earlier in the winter of 2017 compared to that of 2013 (phase angle difference of –52.2º). The greatest difference between peaks in any range was from year 1 to year 3 for depression, with the peak in 2015 occurring 8.5 weeks earlier (phase angle difference of –58.7º) than that in 2013. Schizophrenia and OCD were the only searches to show a positive shift, with peak volume occurring later in the season from year to year. Complete results for each of these comparisons are presented in Table 2.

**Table 2:** Phase angle difference between years within each search term. The value in each cell is the phase angle difference for that year relative to the peak search volume for year 1 (column two). The value in parentheses is the phase angle difference converted to the corresponding number of weeks. A negative value indicates a shift earlier in the year, relative to the first, and a positive value indicates a shift later in the year, relative to the first.

Condition	Peak search, year 1	Year 2, phase angle (weeks)	Year 3, phase angle (weeks)	Year 4, phase angle (weeks)	Year 5, phase angle (weeks)
Anxiety	March, week 3	–31.3º (–4.5)	–44.7º (–6.5)	–48.4º (–7.0)	–42.3º (–6.1)
Autism	April, week 1	–29.8º (–4.3)	–52.7º (–7.6)	–53.9º (–7.8)	–52.2º (–7.5)
Bipolar	March, week 4	–17.2º (–2.5)	–33.3º (–4.8)	–36.2º (–5.2)	–29.5º (–4.3)
Depression	February, week 2	–50.7º (–7.3)	–58.7º (–8.5)	–54.7º (–7.9)	–49.0º (–7.0)
Obsessive-compulsive disorder	January, week 3	29.0º (4.2)	19.5º (2.8)	27.5 (4.0)	38.3º (5.5)
Schizophrenia	January, week 4	41.8º (6.0)	34.0º (4.9)	28.9º (4.2)	27.0º (3.9)
Health	February, week 3	–31.3º (4.5)	–44.7º (6.5)	–48.4º (7.0)	–42.3º (6.1)

## Discussion

The present study was the first to investigate seasonal patterns of Google searches on psychiatric conditions in Ontario, Canada. We found evidence of seasonal patterns for the following search terms and their related queries: “anxiety,” “autism,” “bipolar,” “depression,” “OCD,” “schizophrenia” and “health.” Specifically, we found that winter-to-summer search query differences were maintained for all items with the exception of “suicide” and our general search term (“how”).

In general, our results are in line with previous clinical [11], population [10], or internet [1] studies that report seasonality patterns of mental health issues. Although we did not find seasonal effects for the general, content-agnostic term “how,” our findings demonstrate a similar, increased search interest in health, in general, and mental health terms, in particular, in Ontario during winter months as compared to summer months.

Interestingly, the magnitude of the majority of winter-to-summer search interest peaks in this study were higher than those previously reported for Australia and the United States [1], two large, geographically heterogeneous catchment areas. The difference may be partially accounted for by this study’s focus on Ontario, a single Canadian province that is more geographically homogenous.

The finding of winter peaks and summer troughs in search volumes of general health and mental health terms in Ontario may have several possible explanations. First, it is possible that this finding reflects a general increase in overall internet search activity. Theoretically, a winter decrease in outdoor activity may leave more time for internet searches, in general. There is evidence that Canadians spend less time outdoors in winter than their US counterparts [30]. This hypothesis is not supported, however, by the lack of a seasonality pattern for our general, content-agnostic, search term. Alternatively, it is possible that increases in general health and mental health search volumes in winter reflect decreased happiness in the population as well as increased severity or frequency of health-related issues. A recent study of nearly half-a-billion Twitter and Facebook social media posts [31] reported that cold weather, in particular, was associated with an increase and a decrease in negative and positive expressions of sentiment, respectively. Similarly, there is evidence that cold weather is associated with increased respiratory illness [32] and mortality [33]. Although this is a plausible explanation, it is important to note that the finding of similar temporal patterns for different search terms does not necessarily imply shared or even related underlying risk factors.

Our findings suggest a potential overlap between public interest in OCD and schizophrenia, as interest peaks for the two search terms were almost completely in phase, suggesting very similar peak times during the year. In addition, the mean 5-year seasonal search interest differences were the largest for OCD and schizophrenia terms (45% and 43%, respectively), which were even higher than those for seasonal affective disorder, the prototypical seasonal disorder. Differences in percent change in peak search volumes between OCD and schizophrenia terms were not significant, and the two terms differed significantly from bipolar disorder, depression, and anxiety. These search term similarities are intriguing, given evidence of marked endophenotype differences between OCD and schizophrenia [34]. A possible explanation is that OCD and schizophrenia involve unusual and concerning cognitions and behaviors that are often hard to distinguish from one another [35] and may thus generate overlapping, higher internet search volumes.

We did not observe seasonal patterns of search interest for the term “suicide” and its related searches. Of our mental health-related search terms, “suicide” was the only one that does not pertain to a specific diagnostic category. Indeed, suicidal ideation and suicide rates are associated with multiple risk factors, some of which are not directly related to mental health disorders [36-38] and may have different or absent seasonal patterns. In addition, our findings are in agreement with a recent cross-sectional study in Italy [39] that did not show a statistically significant pattern for suicide and related search terms, resulting in white noise. However, existing studies on the occurrence of suicide have clearly and repeatedly reported a seasonal pattern. For example, a recent systematic review of the temporal distribution of suicide mortality [40] stated that 81.5% of studies of the monthly distribution of suicide monthly peaks corresponded, by study hemisphere, to spring and early summer. Similar results were observed for studies that examined suicide by season [40]. The difference between these findings and our own highlights the complex relationship between suicide-related search volumes and rates of completed suicide or suicide attempts. Solano and colleagues [39] examined the correlation between Google Search and Google News search volumes for the words “suicide,” “to commit suicide,” and “how to commit suicide” and the national suicide rates in Italy between 2008 and 2012. Although the authors reported a significant correlation between searches for “suicide” and suicide rates, the search term lagged suicide attempts by 3 months, raising questions about the nature of the reported association. Furthermore, there was no association between suicide rates and Google searches for “how to commit suicide” or “to commit suicide.” Taken together, our study further highlights the complex association between internet searches on suicide and suicidal behavior, in particular, and internet searches and psychiatric disorders and conditions, in general.

Strengths of the study include its focus on a well-defined geographical area that lacks extreme within-region climate variations; reliance on a 5-year period with weekly data points; and the choice of the continuous wavelet transform, which allowed the isolation and identification of a significant seasonal component for each time series. On the other hand, an important limitation of the study is that the study of internet queries presents unique validation challenges, as seeking information on mental health conditions may not necessarily correspond to actual mental illness in the individual performing the search. In addition, our ability to interpret the results is limited by the lack of demographic information on the sampled population. A third limitation of the present study is that, although there is evidence that the internet has become the most publicly available information search method, Google Trends data do not include absolute numbers. This limitation should be viewed in the context of the ability to quickly track very recent population behavior, which is a clear advantage. Thus, the use of internet query analysis for mental health planning and delivery should be viewed as complementary rather than a replacement for conventional population studies.

In conclusion, our study was the first to focus on mental health searches in Ontario. Seasonal components were detected for all mental health terms, with the exception of “suicide.” Overall, our study demonstrates the feasibility of performing longitudinal tracking of interest in mental health terms in Ontario, a complementary approach to traditional population health studies. Future studies should explore the association between internet search volumes and other online or offline markers of mental health.

## Abbreviations

OCD: obsessive-compulsive disorder
